# Activating the Wnt/β-Catenin Pathway for the Treatment of Melanoma – Application of LY2090314, a Novel Selective Inhibitor of Glycogen Synthase Kinase-3

**DOI:** 10.1371/journal.pone.0125028

**Published:** 2015-04-27

**Authors:** Jennifer M. Atkinson, Kenneth B. Rank, Yi Zeng, Andrew Capen, Vipin Yadav, Jason R. Manro, Thomas A. Engler, Marcio Chedid

**Affiliations:** 1 Oncology Discovery Research, Lilly Research Laboratories, Eli Lilly and Company, Indianapolis, Indiana, 46285, United States of America; 2 Statistics Discovery, Lilly Research Laboratories, Eli Lilly and Company, Indianapolis, Indiana, 46285, United States of America; 3 Discovery Chemistry Research and Technologies, Lilly Research Laboratories, Eli Lilly and Company, Indianapolis, Indiana, 46285, United States of America; University of Kentucky, UNITED STATES

## Abstract

It has previously been observed that a loss of β-catenin expression occurs with melanoma progression and that nuclear β-catenin levels are inversely proportional to cellular proliferation, suggesting that activation of the Wnt/β-catenin pathway may provide benefit for melanoma patients. In order to further probe this concept we tested LY2090314, a potent and selective small-molecule inhibitor with activity against GSK3α and GSK3β isoforms. In a panel of melanoma cell lines, nM concentrations of LY2090314 stimulated TCF/LEF TOPFlash reporter activity, stabilized β-catenin and elevated the expression of Axin2, a Wnt responsive gene and marker of pathway activation. Cytotoxicity assays revealed that melanoma cell lines are very sensitive to LY2090314 in vitro (IC50 ~10nM after 72hr of treatment) in contrast to other solid tumor cell lines (IC50 >10uM) as evidenced by caspase activation and PARP cleavage. Cell lines harboring mutant B-RAF or N-RAS were equally sensitive to LY2090314 as were those with acquired resistance to the BRAF inhibitor Vemurafenib. shRNA studies demonstrated that β-catenin stabilization is required for apoptosis following treatment with the GSK3 inhibitor since the sensitivity of melanoma cell lines to LY290314 could be overcome by β-catenin knockdown. We further demonstrate that in vivo, LY2090314 elevates Axin2 gene expression after a single dose and produces tumor growth delay in A375 melanoma xenografts with repeat dosing. The activity of LY2090314 in preclinical models suggests that the role of Wnt activators for the treatment of melanoma should be further explored.

## Introduction

Melanoma is the sixth most common cancer in the US and patients with metastatic disease have a five year survival rate of less than 20% [1]. Until 2010 the only Food and Drug Administration (FDA) approved therapies for metastatic melanoma were dacabarzine (DTIC) and high-dose interleukin 2, but beginning in 2011 a series of approvals dramatically changed the way unresectable stage III and IV melanomas are treated. The approval of ipilimumab, an immunomodulatory monoclonal antibody directed against the cell surface antigen CTLA-4 and vemurafenib, a targeted agent for the treatment of BRAF^V600E^ [2–4] mutation-positive melanomas paved the way for the subsequent approval of 2 additional agents, trametinib and dabrafenib which together inhibit two distinct components of the RAS/RAF/MEK/ERK pathway and are used in combination to treat melanoma patients with BRAF^V600E^ or BRAF ^V600K^ mutations [5]. Although these approvals certainly represent significant therapeutic options for patients with metastatic melanoma, there are fewer treatment options for patients with non-BRAF mutant melanomas. It is of critical importance to identify therapies which could have potential application in patients with BRAF mutation-negative melanomas and for patients that have failed initial therapy with BRAF inhibitors.

The ‘canonical’ Wnt/β-catenin pathway is heavily implicated in developmental processes and many disease states including cancer. In the absence of Wnt pathway stimulation, β-catenin is sequestered in a ‘destruction complex’ containing the proteins Axin, adenomatous polyposis coli (APC), glycogen synthase kinase-3 (GSK-3), and casein kinase-1 (CK1). Within the complex, CK1 phosphorylates β-catenin at Ser45, which in turn allows for GSK3 phosphorylation of β-catenin at Thr41, Ser37 and Ser33 residues [6]. Phosphorylated β-catenin is next ubiquinated by β-TrCP ubiquitin E3 ligase and degraded by the proteasome [7,8]. In canonical signaling, secreted Wnt glycoproteins bind to specific members of the low-density lipoprotein receptor-related protein (LRP) and Frizzled protein families, activating the pathway. Wnt binding serves to indirectly prevent β-catenin ubiquitination, allowing the destruction complex to become saturated and inactivating the complex [9]. Newly synthesized β-catenin is then able to act as a transcriptional activator by associating with the TCF/LEF family of transcription factors and resulting in the regulation of genes involved in cell proliferation, stem cell maintenance and cell fate determination [10].

The role of the Wnt pathway and β-catenin in melanoma remains controversial with literature both supporting an oncogenic role for Wnt signaling within specific contexts as well as studies indicating that activation of the Wnt pathway in melanoma may be of therapeutic benefit. In numerous tumor types including colorectal and breast cancer, activation of the Wnt/β-catenin pathway is associated with increased tumorigenesis, proliferation and decreased patient survival [11–13]. In contrast, studies in melanoma and prostate cancer indicate that activation of the Wnt/β-catenin pathway, demonstrated by increased nuclear β-catenin, may correlate with an improved rather than poorer prognosis [14–17]. In melanoma, it has been observed that a loss of β-catenin expression occurs with melanoma progression and that nuclear β-catenin levels are inversely proportional to cellular proliferation as measured by Ki67 staining [16–20]. Consistent with this data, a number of studies have sought to understand the role of the Wnt/β-catenin pathway in human melanoma. Chien et al. (2009) demonstrated that overexpression of Wnt3a and subsequent Wnt pathway activation in murine and human melanoma cells resulted in decreased cellular proliferation, increased differentiation and decreased tumor growth in vivo [16]. Similarly, Gallagher et al. (2012) demonstrated that elevated β-catenin levels inhibit melanocyte and melanoma cell line migration in vitro and in vivo. However, they also indicated a role for β-catenin in the stimulation of melanoma metastatic potential when combined with activating mutations in NRAS, indicating that the role of Wnt signaling may be context dependent [21].

More recently, pharmacologic activation of Wnt/β-catenin signaling by Riluzole was reported [22]. Addition of micromolar concentrations of Riluzole, a putative glutamate receptor antagonist (GRM1) enhanced Wnt/β-catenin signaling, shown to decrease proliferation and increase the differentiation of melanoma cells in vitro when combined with exogenous Wnt3a [22]. Similar in vitro observations, including enhancement of Wnt/β-catenin signaling, were also reported when melanoma cells were treated with the non-competitive GRM1 antagonist, BAY 36–7620 [22]. Riluzole was also showed to decrease lymph node metastasis of B16 melanoma cells injected into the footpads of mice [22] collectively suggesting that pharmacological enhancement of Wnt/β-catenin signaling is linked to anti-proliferative/metastatic activity in melanoma cells. We sought to build on this finding in order to better understand if pharmacological activation of the Wnt pathway is worthy of further exploration as a potential therapeutic strategy in the treatment of melanoma. Here we describe a potent, highly selective, small molecule inhibitor of GSK3 capable of activating the Wnt/β-catenin pathway and inducing apoptosis in human melanoma tumor cells at nanomolar concentrations. Melanoma cell lines expressing either mutated RAS or mutant BRAF were equally sensitive to GSK3 inhibition, as were cell lines resistant to the BRAF inhibitor Vemurafenib. In vivo, LY2090314 elevated gene expression of Axin2, a Wnt-responsive gene, and resulted in a tumor growth delay with repeat dosing in both single agent and combination drug studies. Taken together, this data suggests that Wnt pathway activation is a potential therapeutic strategy in patients with BRAF wild type or BRAF mutant melanoma.

## Materials and Methods

### Reagents

The synthesis of LY2090314 was performed as previously described [23]. Vemurafenib was synthesized in house. (2′Z,3′E)-6-Bromoindirubin-3′-acetoxime, BIO-Acetoxime (BIA) was obtained from Chemicon (GSK3 inhibitor X). Riluzole was obtained from Matrix Scientific.

### GSK3 biochemical assay

The inhibitory enzyme activity of LY2090314 was assessed by incubating human recombinant GSK3α or GSK3β in the presence of the peptide substrate YRRAAVPPSPSLSRHSSPHQ(Ps)EDEEE as previously conducted [23].

### Cell Culture

The human melanoma cell lines, A375, M14, Mel1102, A2058, SKMEL2, SKMEL28 and CHL1 were obtained from American Type Culture Collection and cultured as monolayers. MEXF276, MEXF462, MEXF1341 and MEXF1792 patient derived tumor cells were obtained from Oncotest (Freiburg, Germany).

### TOPFlashTCF4/LEF reporter assay

Cells were plated at 250,000 cells/well in 6 well plates and following a 24 hour incubation were transduced with Cignal lentivirus TCF-LEF responsive inducible luciferase reporter according to manufacturer’s instructions (SA Biosciences). Following 72 hours, cells were re-plated and selected for 3 days using puromycin (1μg/ml). Selected cells were seeded in 96 well plates at a density of 10,000 cells/well and allowed to adhere overnight in regular growth media. Following 24 hours, media was removed and replaced with growth media containing 2% fetal bovine serum and the cells exposed to test agents (0.5nmol/L to 1μmol/L) for 5 hours. TCF/LEF activity was assessed using the Bright-glo luciferase substrate reagent according to manufacturer’s instructions (Promega).

### Axin2 gene expression studies

Cells were seeded in 6 well plates at a density of 500,000 cells/well and allowed to adhere overnight in regular growth media. Next, media was replaced and cells treated for a defined time period and defined compound concentration before RNA was isolated using the Qiagen RNeasy kit with a DNAse treatment step according to manufacturer’s instructions (Qiagen). Four micrograms of RNA was used in the subsequent cDNA synthesis reaction using the High Capacity cDNA RT kit according to manufacturer’s instructions (Applied Biosystems). Primer probe sets for Axin2 and GAPDH were obtained from Applied Biosystems and PCR reaction set up using Universal Master Mix in Optical 348 well reaction plates (Applied Biosystems) according to manufacturer’s instructions. Samples were run on the ABI 7900HT (Applied Biosystems) thermocycler and results generated using the standard curve method and SDS2.2.2 software.

For tumor samples, tissue was placed in RNA later (Qiagen) and stored at 4°C prior to processing. At time of processing samples were placed into a Lysing Matrix A tube (Qbiogene) in 0.8ml RLT buffer containing mercaptoethanol (RNeasy kit) and samples homogenized using a Bio101 Fast Prep FP-120 homogenizer at a setting of 5.0 for 30 seconds followed by centrifugation at full speed in a microcentrifuge to pellet debris. Supernatant was collected and the RNeasy Kit (Qiagen) used to process samples according to the manufacturer’s instructions. The remainder of the procedure is as noted above for cells.

### Immunoblotting

Cells were lysed on ice for 15 minutes with lysis buffer containing 1% Triton X100, 25mM Tris pH7.5, 150mM NaCl, 1mM EDTA, 1mM EGTA and 1x Halt protease and phosphatase inhibitor cocktail (Thermo scientific). Lysates were freeze-thawed before being scraped and sonnicated. Protein lysate supernatant was collected by centrifugation at full speed in a microcentrifuge for 10 minutes at 4°C. Equal amounts of protein were resolved by 4–20% SDS-PAGE gels (Biorad) and the iblot system was used to transfer protein to a nitrocellulose membrane (Invitrogen). Nonspecific antibody binding was blocked in a 5% milk solution for 1 hour at room temperature. Antibodies used in the studies were sourced from the following vendors: β-catenin (Cell Signaling, Cat. #9562), pβ-Catenin (serine 33/37, threonine 41) (Cell Signaling, Cat. # 9561), GAPDH (Cell Signaling, Cat. # 2118), Axin1 (Cell Signaling, Cat. # 2087), Axin2 (Cell Signaling, Cat. # 2151), pJNK (Cell Signaling, Cat. # 9251) and pMEK (Cell Signaling, Cat. # 9121). The pGSK3 primary antibody, which recognizes pTyr 279-GSK3α and pTyr 216-GSK3β was obtained from Abcam (Cat. # 52188). Antibodies were incubated with membrane overnight at 4°C at 1:1000 dilution in 1% BSA before the membranes were washed and probed with a donkey anti-Rabbit horseradish peroxidase-conjugated secondary antibody (GE Healthcare). Antibody reactivity was detected by chemiluminescence using Super-signal West Femto substrate (Pierce).

### Cell chemosensitivity and caspase activation assay

Cells were seeded in 96 well plates at a density of 2,000 cells/well and allowed to adhere overnight in regular growth media. Following 24 hours, media was removed and replaced with growth media containing 2% fetal bovine serum and the cells exposed to test agents (0.5nmol/L to 10μmol/L) for 72 hours. In vitro chemosensitivity of melanoma cells to LY2090314, BIA and Vemurafenib was determined using the CellTiter-Glo assay according to manufacturer’s instructions (Promgea). Caspase 3/7 activation was determined using the Caspase-Glo assay according to manufacturer’s instructions (Promega). Nonlinear regression and sigmoidal dose-response curves were used to calculate the half maximal inhibitory concentration (IC_50_) using GraphPad Prism 6 software.

### PARP cleavage assay

Cells were seeded in 6 well plates at a density of 250,000 cells/well and allowed to adhere overnight in regular growth media. Following 24 hours, media was replaced with fresh growth medium containing LY2090314 (final concentration 5nM, 15nM) and the cells exposed to test agents for 72 hours. PARP cleavage was assessed per 50μg total protein using the Pathscan Cleaved PARP ELISA (Asp214) sandwich ELISA kit according to manufacturer’s instructions (Cell Signaling Technology). Levels of cleaved PARP were calculated relative to DMSO control treated cells.

### Immunoprecipitation

In order to assess ‘free’ β-catenin within cell lysates before and after treatment with LY2090314, we measured the amount of β-catenin available for binding to the C-terminal region of E-cadherin fused to a FLAG tag. This method has been described previously [24]. Briefly, lysates representing 300μg total protein were incubated with 10μg His-Flag-E-cadherin. Anti-FLAG M2 Affinity gel (Sigma) was washed and incubated with the lysate/E-cadherin mix overnight at 4°C in order to bind E-cadherin/β-catenin complexes. Following the overnight incubation, the resin was washed 3 times with lysis buffer before being resuspended in gel loading buffer (Boston biosciences) containing β-mercaptoethanol. Samples were centrifuged at 8000g and supernatants used for immunoblot analysis.

### shRNA knockdown of β-catenin

A375 and M14 cells were plated at a density of 250,000 cells/well in a 6 well plate in complete culture media. After 24 hours, cells were transduced with Mission lentiviral particles (Sigma Adrich) targeting β-catenin, GSK3α, GSK3β, Axin1 or control at an MOI of 2 in the presence of polybreen (8μg/ml). Following a further 24 hours, infection media was replaced with 2ml complete culture medium and the cells selected using puromycin (2μg/ml) after a further 48 hours. Protein knockdown was verified by immunoblotting and the cells maintained in 2ug/ml puromycin.

### LY2090314 pharmacokinetic studies

All animal work was performed in an Association for Assessment of Laboratory Animal Care (AALAC)-certified facility and was approved by Eli Lilly and Company Institutional Animals Care and Use committee. To understand the plasma pharmacokinetics of LY2090314 in vivo following i.v. administration, CD1 nu/nu non-tumor bearing mice (Harlan, Indianapolis, IN) were injected i.v. with 5mg/kg LY2090314 and blood collected by cardiac puncture at the times indicated (5, 15, 30, 60 120 minutes post dose). Mice were sacrificed using isoflurane and cervical dislocation. Blood was centrifuged at 5,000 x g for 10 minutes, and the resulting plasma analyzed for drug concentration using HPLC and mass spectrometry.

### In vivo studies

Five million A375 human melanoma cancer cells were injected S.C. in the flank of female 6 to 8 week old athymic nude mice (Harlan, Indianapolis, IN) in a 1:1 mixture with matrigel (Becton Dickinson, Bedford, MA). Mice were monitored daily for palpable tumors. When tumors reached ~100mm^2^ mice were randomized into groups receiving either LY2090314 (25 mg/kg Q3D) or vehicle (20% Captisol/0.01N HCl) via i.v. administration. Tumor volume (measured by calipers) and animal body weight were recorded twice weekly. Tumor volumes were calculated using the formula: (a^2^ x b)/2 (a being the smaller and b being the larger dimension of the tumor). For combination studies with DTIC (60 mg/kg QD), LY2090314 was dosed at 2.5 mg/kg Q3D and tumor growth monitored.

For in vivo target inhibition studies in xenograft tissue LY2090314 (25mg/kg) was administered to mice harboring A375 tumors approximately 200mm^2^ in volume and tumor tissue collected for RNA expression analysis at 1, 2, 4, 6, 8 and 24hours postdose.

### Statistical analysis

In vitro concentration response curves were fit and IC_50_ estimates were calculated using Graphpad Prism (Version 6). A mixed effects repeated measures ANOVA model was used to analyze the tumor growth studies. Tumor volume data were transformed to the log scale to account for heterogeneous variance across time and treatment groups. The repeated measures ANOVA model was fit using the MIXED procedure in SAS (version 9.3, Cary, NC). This procedure was used to estimate least squares means and standard errors for treatment by time as well as p-values for comparisons between treatments within days. This same model was also used to estimate the drug combination effect of LY2090314 and dacarbazine. This was done by estimating the 2x2 interaction effect and p-value from a contrast consisting of the vehicle control, each single agent and the combination group.

## Results and Discussion

### LY2090314 is a potent, selective GSK3 inhibitor which activates the Wnt pathway in melanoma cell lines

**Fig 1:**
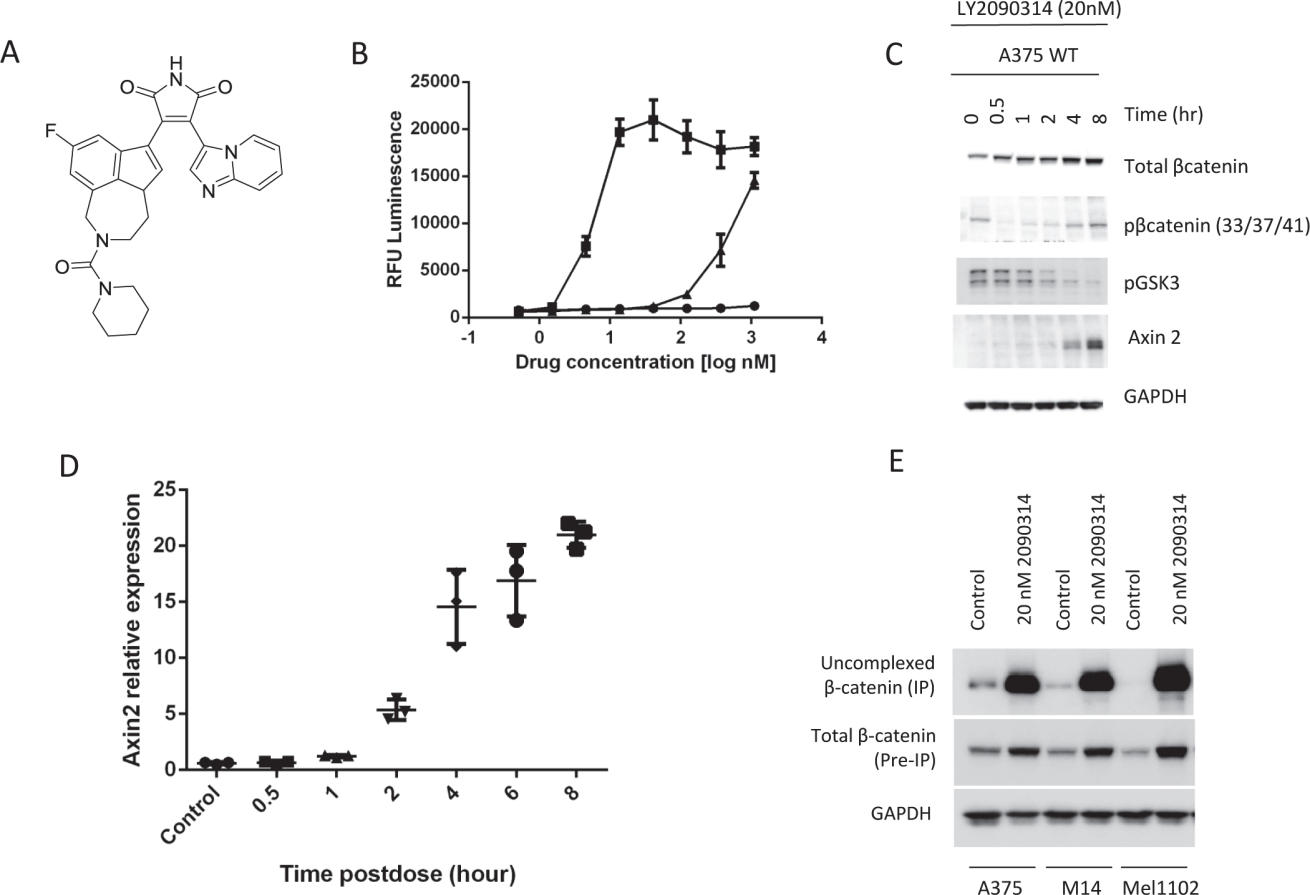
LY2090314 is a GSK inhibitor which elevated Wnt signaling in melanoma cell lines. **A**. Structure of LY2090314. **B**. Riluzole (**▲**), LY2090314 (■) and BIA (●) activated the TCF/LEF luminescent reporter in A375 cells following 5 hours of drug exposure. **C**. LY2090314 (20nM) decreased phosphorylation of GSK3α/β in A375 cells and increased β-catenin and Axin2 protein levels as determined by Western blot. **D**. LY2090314 (20nM) treatment of A375 cells increased Axin2 gene expression in a time dependent manor. **E**. Uncomplexed β-catenin IP experiments reveal a large increase in β-catenin protein following LY2090314 (20nM) treatment for 48 hours.

In order to pharmacologically activate the Wnt pathway in melanoma cells without the need for exogenous Wnt3a, and therefore better assess activation of the Wnt pathway as a treatment strategy using small molecule therapeutics, we sought to investigate a highly potent activator of Wnt signaling. LY2090314 is a small molecule bisarylmaleimide (Fig 1A) with in vitro activity against both human recombinant GSK3α (IC50 1.5nM) and GSK3β (IC50 0.9nM) in a cell free assay. LY2090314 is highly selective towards GSK3 as demonstrated by its fold selectivity relative to a large panel of kinases (S1 Fig). We assessed the ability of LY2090314, Riluzole and BIA (a frequently utilized and commercially available GSK3 inhibitor) to stimulate TCF/LEF TOPFlash activity in A375 melanoma cells. Consistent with activation of Wnt signaling, LY2090314 stimulated the TCF/LEF TOPFlash reporter at low nanomolar concentrations (Fig 1B) while BIA was able to increase reporter activity at high nanomolar concentrations. In contrast, and consistent with the literature, Riluzole was a poor inducer of the TCF/LEF reporter activity in the absence of Wnt3a conditioned media [22]. To investigate whether LY2090314 functionally activates Wnt signaling we characterized its ability to stabilize β catenin and to stimulate the expression of Axin2 in A375 melanoma cells. As Western blotting demonstrates, treatment with 20nM LY2090314 promotes a time-dependent stabilization of β-catenin total protein as well as an induction of Axin2 (Fig 1C). Treatment with LY2090314 also resulted in inhibition of GSK3 phosphorylation at tyrosine 279 and β-catenin at serines 33/37 and threonine 41. Gene expression analysis in A375 cells confirmed the time and dose dependent increase in Axin2 mRNA expression following LY2090314 treatment (Fig 1D and S2 Fig). To further analyze the effect of GSK3 inhibition on the levels of free β-catenin we performed E-cadherin IP experiments to isolate ‘uncomplexed’ β-catenin from cells [24]. This allowed us to assess changes in the amount of β-catenin protein in the cell, free to participate in Wnt signaling. As Fig 1E demonstrates, treatment with LY2090314 resulted in a large increase in uncomplexed β-catenin, a finding compatible with Wnt signaling activation. Taken together, these experiments demonstrate that LY2090314 is able to potently stabilize β-catenin protein and activate Wnt signaling in vitro. Our data also indicates that LY2090314 is at least 10–100 fold more potent than either BIA or Riluzole in its ability to activate Wnt signaling [25].

### LY2090314 potently induces apoptotic cell death in a panel of melanoma cell lines irrespective of BRAF mutation status

**Fig 2:**
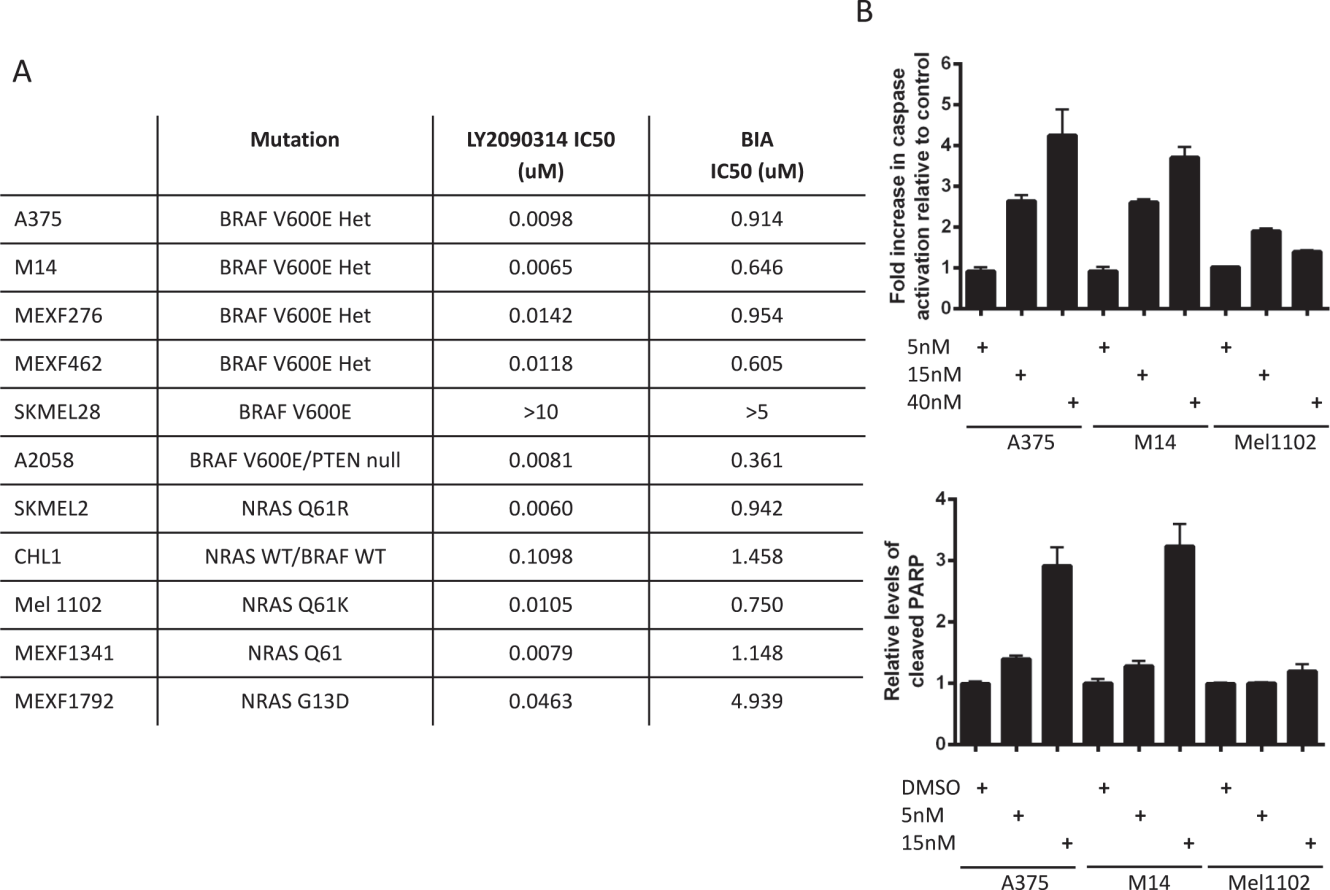
LY2090314 potently induces apoptotic cell death in a range of melanoma cell lines irrespective of BRAF mutation status. **A**. 72hr cytotoxicity assays (CellTiter-Glo) reveal LY2090314 activity in the nanomolar range in numerous melanoma cell lines. The commercially available GSK3 inhibitor BIA demonstrates activity in the micromolar range. **B**. Cleaved caspase assays (Promega) reveal an induction of cleaved caspase3/7 prior to cell death in melanoma cell lines, data presented is following 72 hours LY2090314 drug treatment. **C.** Cleaved PARP can be detected prior to cell death following cell treatment with LY2090314 indicating that LY2090314 induces cell death via apoptosis.

To test the impact of GSK3 inhibition on the proliferation of human melanoma, a panel of 11 cell lines representing both BRAF WT/NRAS mutant and BRAF mutant cells were subjected to 72 hour proliferation assays in the presence of LY2090314 or BIA (Fig 2A). LY2090314 demonstrated a higher potency in all cell lines relative to BIA and was active in the nanomolar range in 10/11 cell lines tested. Mutation status in terms of BRAF and NRAS, the most commonly found mutations in human melanoma [3], did not have a significant effect on the ability of cells to respond to LY2090314. The apoptosis markers of caspase 3/7 activation and PARP cleavage were assessed to associate the results from the proliferation assay to apoptotic cell death. Following 72hr drug exposure both activated caspase 3/7 (Fig 2B) and cleaved PARP (Fig 2C) were induced in the single digit nanomolar range by drug treatment indicating that LY2090314 induces cell death via apoptosis. The antiproliferative effects of LY2090314 observed in melanoma cell lines was not evident in a variety of other solid tumor lines tested (S1 Table) suggesting that LY2090314 is not a general cytotoxic agent and in line with the concept that activation of the Wnt pathway is associated with reduced proliferation of melanoma cells.

### Cell death induced by LY2090314 is dependent upon β-catenin and Wnt signaling

**Fig 3:**
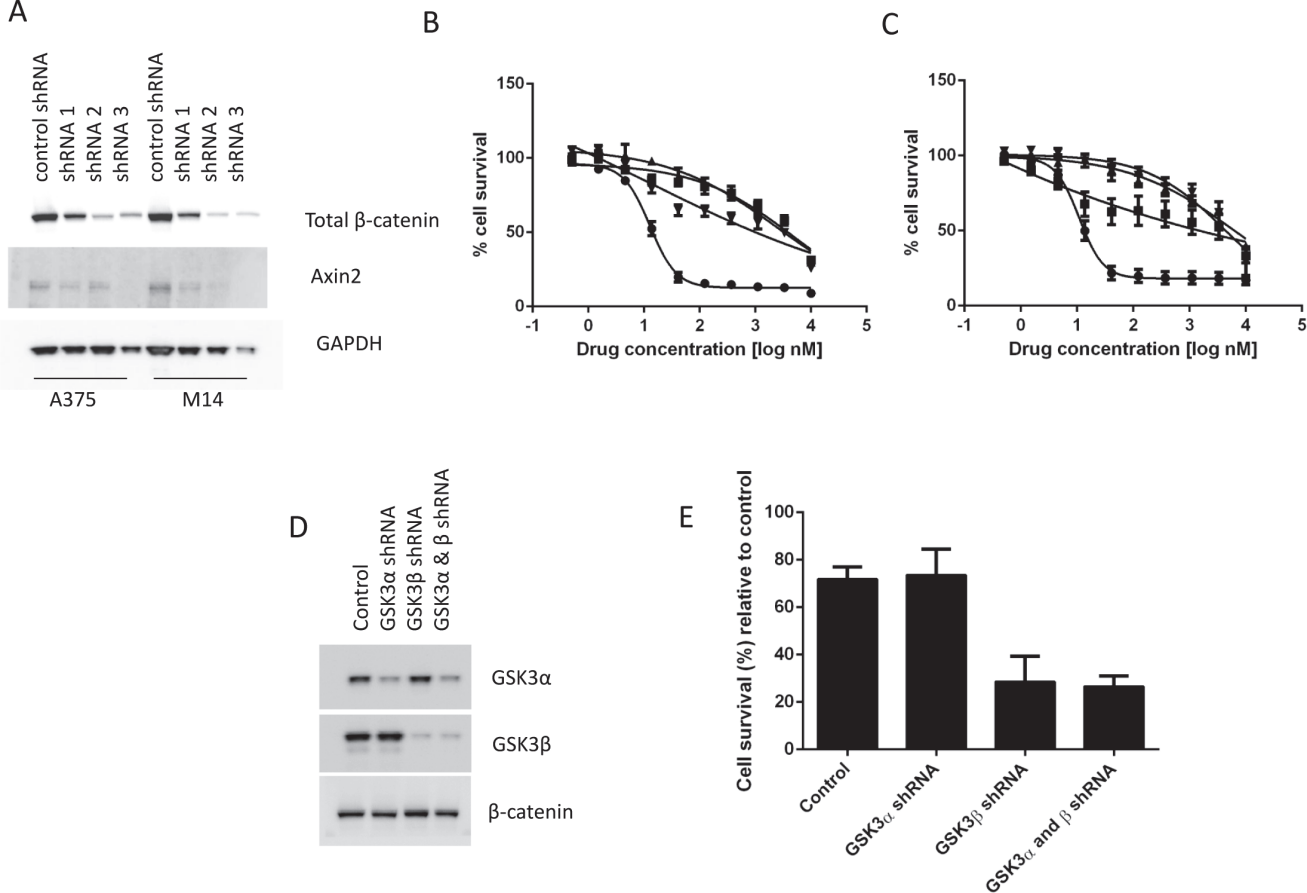
Cell death induced by LY2090314 is dependent on β-catenin and GSK3β knockdown increases the sensitivity of cells to LY2090314. **A**. Melanoma cells stably transfected with shRNAs targeting β-catenin display decreased β-catenin and Axin2 protein expression by western blot. A375 (**B**) and M14 (**C**) cells expressing shRNAs targeting β-catenin (● Control; ■ β-catenin shRNA 1; **▲** β-catenin shRNA 2; ▼ β-catenin shRNA 3) become resistant to LY2090314 suggesting that β-catenin is required for apoptotic cell death in response to LY2090314. **D, E.** A375 cells targeted with GSK3β shRNA, but not GSK3α shRNA, demonstrates increased sensitivity to LY2090314 (4.5nM, 72hr).

To further investigate the role of Wnt activation, and more specifically β-catenin stabilization in LY2090314-mediated cell death, we assessed the impact of β-catenin knockdown in melanoma cells. Protein knockdown experiments using lentiviral vectors containing shRNAs targeting β-catenin were used to infect A375 and M14 cells, both sensitive to apoptosis induction after treatment with LY2090314. β-catenin knockdown (Fig 3A) rendered both cell lines resistant to the anti-proliferative effects of LY2090314, as noted by a significant increase in EC50 (Fig 3B and 3C), demonstrating that β-catenin is required for the apoptotic cell death resulting from GSK3 inhibition. In addition to β-catenin knockdown, overexpression of the dominant negative form of TCF4, which activates Wnt signaling by directly binding cytosolic β-catenin and facilitating its translocation to the nucleus [26], also rendered the cells resistant to the anti-proliferative effects of LY2090314 (S3 Fig), again indicating the role of the Wnt pathway in controlling response to GSK3 inhibition in melanoma. In line with previous observations [27], shRNAs targeting of GSK3α and GSK3β had minimal adverse effects on cell proliferation (data not shown) however, GSK3β, but not GSK3α, knockdown cells were more sensitive to LY2090314 (Fig 3D and 3E). Together, these findings suggest that impairment of LY2090314-mediated Wnt signaling activation leads to decreased apoptosis in melanoma cells. Since the effect of LY2090314 on cell proliferation was rescued by shRNAs targeting β-catenin, this indicates that β-catenin is an important mediator of apoptosis in melanoma cells in response to LY2090314, a finding that further supports and extends prior studies which demonstrate that over expression of Wnt3a could reduce tumor growth in a murine melanoma model [16].

It is known that GSK3α and GSK3β are structurally highly conserved proteins and previous studies have demonstrated that both GSK3 isoforms perform a redundant role in Wnt/β-catenin signaling [28]. Interestingly, our data showed that GSK3β, but not GSK3α knockdown cells were more sensitive to the apoptotic activity of LY2090314 in melanoma cells, potentially suggesting that additional, non Wnt pathway related mechanisms may contribute to the activity of LY2090314 in melanoma cells. Although LY was shown to be selective for GSK3 when tested against a large kinase panel (S1 Fig), off-target effects cannot be conclusively ruled out at this time.

### LY2090314 remains active in cell lines resistant to Vemurafenib and has an independent mechanism of action

**Fig 4:**
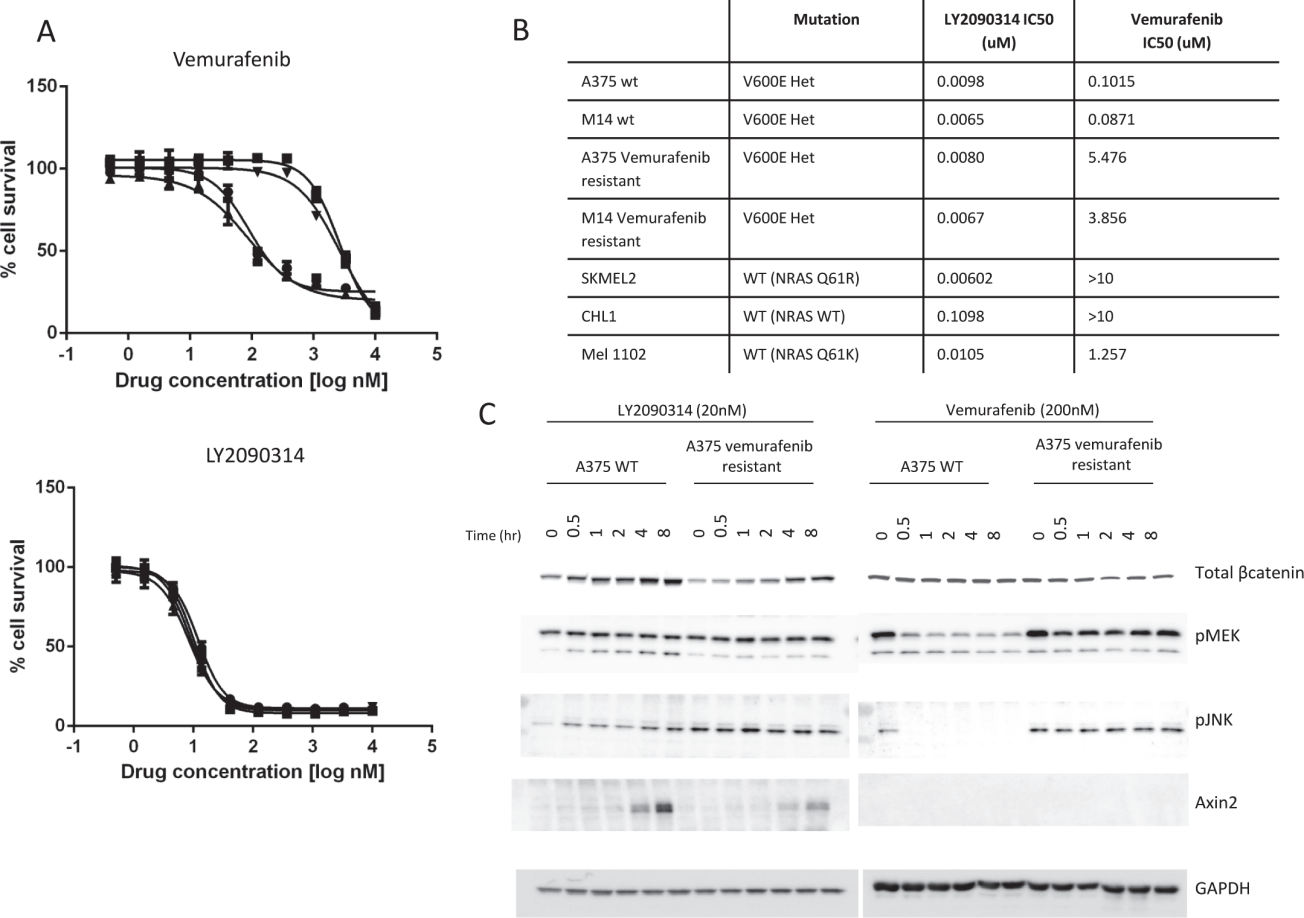
LY2090314 demonstrates activity in cell lines resistant to the BRAF inhibitor Vemurafenib and has an independent mechanism of action. **A**. Wild type melanoma cell lines M14 and A375 are sensitive to growth inhibition by Vemurafenib relative to cells selected as resistant to Vemurafenib. However, LY2090314 retains potency in both wild type and Vemurafenib resistant cell lines (● A375 control; ■ Vemurafenib resistant A375; ▲ M14 control; ▼ Vemurafenib resistant M14). **B**. In a panel of melanoma cell lines, variable sensitivity to Vemurafenib can be observed using 72 hr cytotoxicity assays whilst all cells tested displayed sensitivity to LY2090314. **C**. LY2090314 and Vemurafenib have distinct mechanisms of action. Following drug treatment, cells were analyzed for compound effect on the Wnt and Ras pathways and demonstrate differential signaling pathway modulation.

Since melanoma patients harboring BRAF mutations rapidly become resistant to Vemurafenib, we questioned if Vemurafenib resistant cells retain sensitivity to LY2090314. Using M14 and A375 cells selected for by growth in Vemurafenib over a 3 month period we observed a significant shift in the IC_50_ of Vemurafenib in selected cells whilst no change in the sensitivity to LY2090314 was observed (Fig 4A) [29]. A summary of IC_50_ values for Vemurafenib and LY2090314 in sensitive and resistant cells is shown in Fig 4B. To monitor the status of the Wnt and MAPK signaling pathways upon treatment with Vemurafenib or LY2090314, we treated A375 (Fig 4C) or M14 (S4 Fig) parental and Vemurafenib-resistant cells with compound for up to 8 hours followed by Western blot analysis of protein expression. As Fig 4C demonstrates, treatment with LY2090314 increased β-catenin protein levels and elevated Axin2 in both parental and Vemurafenib-resistant A375 cells. pMEK remained unchanged in cells treated with LY2090314 while pJNK was observed to moderately increase in parental A375 cells. JNK is most notably involved in the non-canonical Wnt pathway [30] although β-catenin has also been described as a binding partner of c-Jun with a role in JNK signaling [31]. In contrast, Vemurafenib had little effect on β-catenin or Axin2 protein levels but rather blocked the expression of pJNK and pMEK in parental cells, consistent with the predicted mechanism of action. As published, in the Vemurafenib resistant cells we observe MAPK pathway reactivation and therefore do not detect significant changes in pJNK or pMEK following Vemurafenib treatment [29]. These experiments indicate non-overlapping mechanisms of action for LY2090314 and the BRAF inhibitor Vemurafenib and suggest that GSK3 inhibition may be a potential therapeutic strategy in melanoma patients who fail therapy with a BRAF inhibitor. Previous studies by other groups suggest interplay between the RAS/RAF/MEK pathway and the Wnt pathway in melanoma [32]. Biechele et al. (2012) report significant synergy between the BRAF inhibitor PLX4032 and Wnt3a conditioned media in inducing cellular apoptosis in melanoma models. In our system we were unable to demonstrate significant synergy between Wnt3a or LY2090314 and Vemurafenib in vitro or in vivo (data not shown), a difference which cannot be easily explained. The authors also indicated that the expression level of β-catenin and Axin1 is an important determinant in the cellular response to BRAF inhibition but in our hands neither β-catenin nor Axin1 knockdown via shRNAs had any effect on the cellular response to Vemurafenib in A375 or M14 cells (S5 and S6 Figs) [32]. Together, our data supports the activation of independent signaling pathways with LY2090314 and Vemurafenib. Crucially, whilst we fail to demonstrate an association between BRAF inhibitor sensitivity and Wnt pathway activation, both our current study and previous publications support the notion that Wnt pathway activation is a potential strategy in the treatment of human melanoma [27,32].

### LY2090314 demonstrates single agent activity in the A375 melanoma model and synergizes with DTIC in vivo

**Fig 5:**
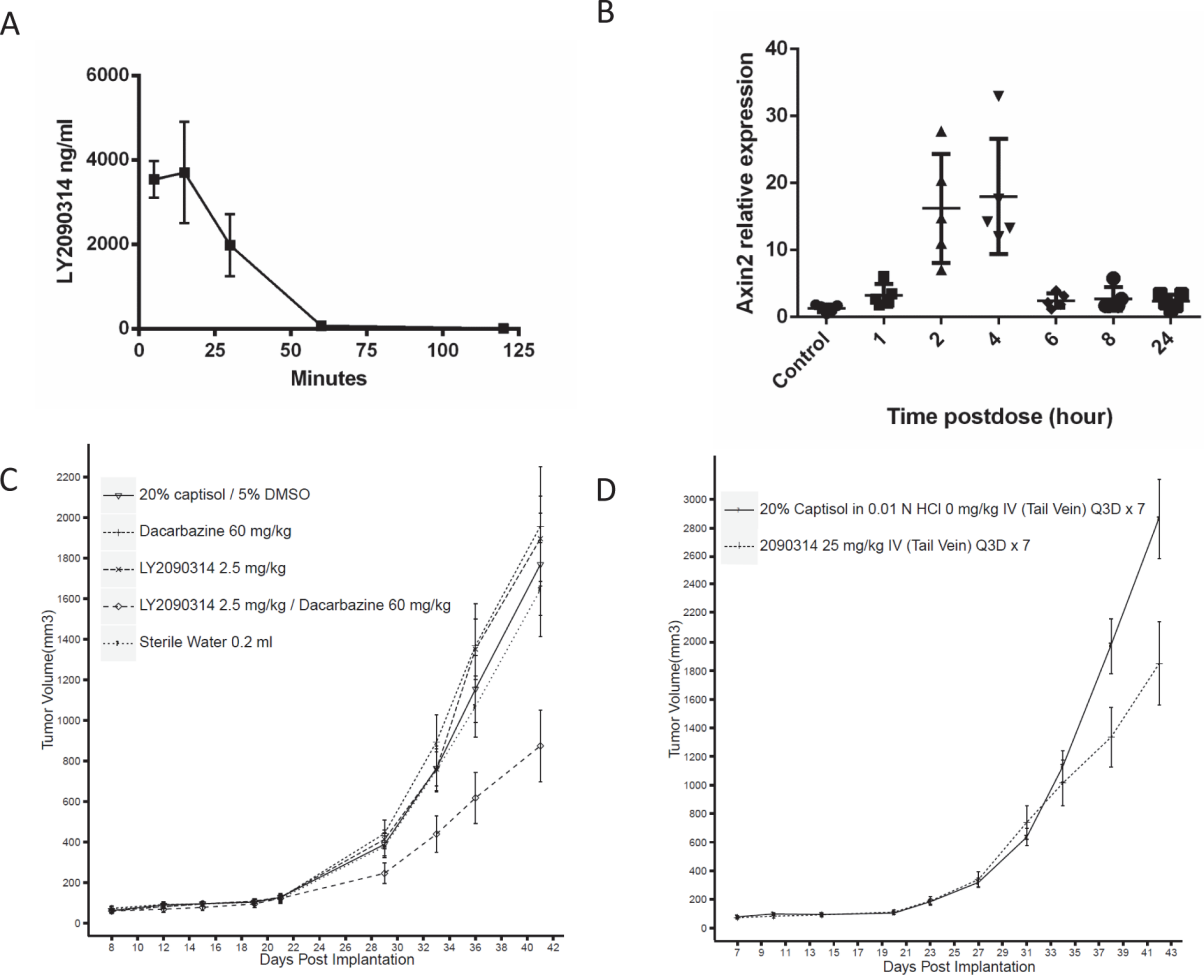
LY2090314 elevates Axin2 gene expression in vivo, demonstrates single agent activity in the A375 xenograft model of melanoma and enhances the efficacy of DTIC. **A**. Plasma PK of LY2090314 in non-tumor bearing mice. **B**. A single dose of LY2090314 (25mg/kg) elevates Axin2 gene expresion in vivo, a marker of wnt pathway activation. **C**. Subcutanous A375 xenografts were treated with 25mg/kg LY2090314 Q3D and resulted in a significant tumor growth delay (p<0.006). **D**. LY2090314 (2.5mg/kg Q3D) enhances the efficacy of DTIC (60mg/kg QD) in A375 xenografts (p< 0.02).

We sought to assess the ability of LY2090314 to activate the Wnt pathway in vivo and subsequently question if pathway elevation could lead to antitumor efficacy in melanoma. In mouse, LY2090314 is rapidly cleared and has a plasma half-life of 36 minutes (Fig 5A). In studies assessing the in vivo gene expression of Axin2, a Wnt responsive gene, we observed a significant induction of Axin2 mRNA at 2 and 4 hours post dose of LY2090314 in A375 xenograft tumor tissue (Fig 5B). This finding is in agreement with our in vitro experiments which also reveal Axin2 elevation 2–4 hours after initial drug exposure (Fig 1E). The rapid decline in Axin2 gene expression after 4 hours is consistent with the short half-life and pharmacokinetic properties of the compound in vivo (Fig 5A and 5B). Despite the transient elevation of the Wnt pathway with LY2090314 treatment, we were able to observe single agent antitumor efficacy in subcutaneous A375 xenografts dosed every 3 days (Fig 5C, p<0.003). In addition, we explored the ability of LY2090314 to synergize with DTIC in vivo and observed that the combination treatment displayed statistically significant greater than additive effects relative to control and single treatment groups (Fig 5D, p<0.02). In these studies we did not detect significant animal weight loss or other clinical signs. It is important to note that caution should be adopted when exploring the potential use of Wnt activators in cancer therapy due to their ability to increase the proliferation of normal tissues. Optimization of compound dosing and scheduling will be of vital importance when determining if compounds such as these have a sufficient therapeutic window for the treatment of melanoma. The studies presented here provide proof-of-concept data supporting the use of Wnt activators in the treatment of melanoma and support further investigation of GSK3 inhibitors for melanoma therapy with particular attention given to the effects on healthy tissues.

## Conclusion

Here we describe a selective small molecule GSK3 inhibitor with potent in vitro activity against GSK3α and GSK3β with the ability to rapidly induce β-catenin dependent apoptosis in preclinical melanoma models. Both tumors driven by RAS and those driven by BRAF responded to LY2090314 suggesting that Wnt activation is a potential therapeutic strategy in both groups of patients. LY2090314 demonstrated in vivo activity as both a single agent and in combination with DTIC. The in vitro and in vivo activity of LY2090314 in preclinical models suggests that the role of Wnt activators for the treatment of both BRAF and NRAS driven human melanoma should be further explored.

## Supporting Information

S1 Fig LY2090314 selectivity profile. Single point (20uM) percentage inhibition studies were performed for 200 kinases and IC50 experiments conducted for 44 enzymes. Fold selectivity relative to GSK3β is represented in the figure.
(PDF)

S2 Fig LY2090314 treatment of A375 cells increased Axin2 gene expression in a dose dependent manor. (PDF)

S3 Fig A dominant/negative TCF4 construct renders cells insensitive to LY2090314. Dominant/negative TCF4 was delivered to cells using lentivirus. Following selection, cytotoxicity assays were performed with LY2090134 according to materials and methods (● A375 control; ■ A375 TCF4 DN; ▲ M14 control; ▼ M14 TCF4 DN).
(PDF)

S4 Fig LY2090314 and vemurafenib have distinct mechanisms of action. Following drug treatment, cells were analyzed for compound effect on the Wnt and Ras pathways and demonstrate differential signaling pathway modulation.
(PDF)

S5 Fig Cell death induced by LY2090314 is not dependent on Axin1. A. Melanoma cells stably transfected with shRNAs targeting Axin1 display decreased Axin1 protein expression by western blot. A375 (B) and M14 (C) cells expressing shRNAs targeting Axin1(● Control; ■ Axin1 shRNA 1; ▲ Axin1 shRNA 2; ▼ Axin1 shRNA 3) retain sensitivity to LY2090314 suggesting Axin1 does not play a role in the apoptotic response to compound treatment.
(PDF)

S6 Fig Cell death induced by Vemurafenib is not dependent on β-catenin. Melanoma cells stably transfected with shRNAs targeting β-catenin display decreased β-catenin protein expression by western blot (Fig 3). A375 (A) and M14 (B) cells expressing shRNAs targeting β-catenin(● Control; ■ β-catenin shRNA 1; ▲ β-catenin shRNA 2; ▼ β-catenin shRNA 3) retain sensitivity to vemurafenib suggesting β-catenin does not play a role in the apoptotic response to compound treatment.
(PDF)

S1 Table LY2090134 relative IC50 values in a panel of human tumor cell lines. Cell lines were treated for 72 hours in an exponential phase of growth and the IC50 determined using a cell viability method, CellTiter-Glo (see materials and methods).
(PDF)

## References

[1] Jemal A, Bray F, Center MM, Ferlay J, Ward E, Forman D (2011) Global cancer statistics. CA Cancer J Clin 61: 69–90. 10.3322/caac.20107 21296855

[2] FDA (2011) FDA approves Zelboraf and companion diagnostic test for late-stage skin cancer In: Jefferson E, editor. http://www.fda.gov/NewsEvents/Newsroom/PressAnnouncements/ucm268241.htm: FDA.

[3] Hodis E, Watson IR, Kryukov GV, Arold ST, Imielinski M, Theurillat JP, et al (2012) A landscape of driver mutations in melanoma. Cell 150: 251–263. 10.1016/j.cell.2012.06.024 22817889 PMC3600117

[4] Wagle N, Emery C, Berger MF, Davis MJ, Sawyer A, Pochanard P, et al (2011) Dissecting therapeutic resistance to RAF inhibition in melanoma by tumor genomic profiling. J Clin Oncol 29: 3085–3096. 10.1200/JCO.2010.33.2312 21383288 PMC3157968

[5] Flaherty KT, Infante JR, Daud A, Gonzalez R, Kefford RF, Sosman J, et al (2012) Combined BRAF and MEK inhibition in melanoma with BRAF V600 mutations. N Engl J Med 367: 1694–1703. 10.1056/NEJMoa1210093 23020132 PMC3549295

[6] Liu C, Li Y, Semenov M, Han C, Baeg GH, Tan Y, et al (2002) Control of beta-catenin phosphorylation/degradation by a dual-kinase mechanism. Cell 108: 837–847. 11955436 10.1016/s0092-8674(02)00685-2

[7] Aberle H, Bauer A, Stappert J, Kispert A, Kemler R (1997) beta-catenin is a target for the ubiquitin-proteasome pathway. EMBO J 16: 3797–3804. 9233789 10.1093/emboj/16.13.3797 PMC1170003

[8] Kitagawa M, Hatakeyama S, Shirane M, Matsumoto M, Ishida N, Hattori K, et al (1999) An F-box protein, FWD1, mediates ubiquitin-dependent proteolysis of beta-catenin. EMBO J 18: 2401–2410. 10228155 10.1093/emboj/18.9.2401 PMC1171323

[9] Li VS, Ng SS, Boersema PJ, Low TY, Karthaus WR, Gerlach JP, et al (2012) Wnt signaling through inhibition of beta-catenin degradation in an intact Axin1 complex. Cell 149: 1245–1256. 10.1016/j.cell.2012.05.002 22682247

[10] Clevers H, Nusse R (2012) Wnt/beta-catenin signaling and disease. Cell 149: 1192–1205. 10.1016/j.cell.2012.05.012 22682243

[11] Scholer-Dahirel A, Schlabach MR, Loo A, Bagdasarian L, Meyer R, Guo R, et al (2011) Maintenance of adenomatous polyposis coli (APC)-mutant colorectal cancer is dependent on Wnt/beta-catenin signaling. Proc Natl Acad Sci U S A 108: 17135–17140. 10.1073/pnas.1104182108 21949247 PMC3193196

[12] Liu CC, Prior J, Piwnica-Worms D, Bu G (2010) LRP6 overexpression defines a class of breast cancer subtype and is a target for therapy. Proc Natl Acad Sci U S A 107: 5136–5141. 10.1073/pnas.0911220107 20194742 PMC2841938

[13] Anastas JN, Moon RT (2013) WNT signalling pathways as therapeutic targets in cancer. Nat Rev Cancer 13: 11–26. 10.1038/nrc3419 23258168

[14] Fattet S, Haberler C, Legoix P, Varlet P, Lellouch-Tubiana A, Lair S, et al (2009) Beta-catenin status in paediatric medulloblastomas: correlation of immunohistochemical expression with mutational status, genetic profiles, and clinical characteristics. J Pathol 218: 86–94. 10.1002/path.2514 19197950

[15] Horvath LG, Henshall SM, Lee CS, Kench JG, Golovsky D, Brenner PC, et al (2005) Lower levels of nuclear beta-catenin predict for a poorer prognosis in localized prostate cancer. Int J Cancer 113: 415–422. 15455387 10.1002/ijc.20599

[16] Chien AJ, Moore EC, Lonsdorf AS, Kulikauskas RM, Rothberg BG, Berger AJ, et al (2009) Activated Wnt/beta-catenin signaling in melanoma is associated with decreased proliferation in patient tumors and a murine melanoma model. Proc Natl Acad Sci U S A 106: 1193–1198. 10.1073/pnas.0811902106 19144919 PMC2626610

[17] Kageshita T, Hamby CV, Ishihara T, Matsumoto K, Saida T, Ono T (2001) Loss of beta-catenin expression associated with disease progression in malignant melanoma. Br J Dermatol 145: 210–216. 11531781 10.1046/j.1365-2133.2001.04336.x

[18] Lucero OM, Dawson DW, Moon RT, Chien AJ (2010) A re-evaluation of the "oncogenic" nature of Wnt/beta-catenin signaling in melanoma and other cancers. Curr Oncol Rep 12: 314–318. 10.1007/s11912-010-0114-3 20603725 PMC2910886

[19] Arozarena I, Bischof H, Gilby D, Belloni B, Dummer R, Wellbrock C (2011) In melanoma, beta-catenin is a suppressor of invasion. Oncogene 30: 4531–4543. 10.1038/onc.2011.162 21577209 PMC3160497

[20] Sinnberg T, Menzel M, Ewerth D, Sauer B, Schwarz M, Schaller M, et al (2011) beta-Catenin signaling increases during melanoma progression and promotes tumor cell survival and chemoresistance. PLoS One 6: e23429 10.1371/journal.pone.0023429 21858114 PMC3157382

[21] Gallagher SJ, Rambow F, Kumasaka M, Champeval D, Bellacosa A, Delmas V, et al. (2012) Beta-catenin inhibits melanocyte migration but induces melanoma metastasis. Oncogene. 10.1038/onc.2012.229 PMC3637425 22665063

[22] Biechele TL, Camp ND, Fass DM, Kulikauskas RM, Robin NC, White BD, et al (2010) Chemical-genetic screen identifies riluzole as an enhancer of Wnt/beta-catenin signaling in melanoma. Chem Biol 17: 1177–1182. 10.1016/j.chembiol.2010.08.012 21095567 PMC3044442

[23] Engler TA, Henry JR, Malhotra S, Cunningham B, Furness K, Brozinick J, et al (2004) Substituted 3-imidazo[1,2-a]pyridin-3-yl- 4-(1,2,3,4-tetrahydro-[1,4]diazepino-[6,7,1-hi]indol-7-yl)pyrrole-2,5-diones as highly selective and potent inhibitors of glycogen synthase kinase-3. J Med Chem 47: 3934–3937. 15267232 10.1021/jm049768a

[24] Bafico A, Gazit A, Wu-Morgan SS, Yaniv A, Aaronson SA (1998) Characterization of Wnt-1 and Wnt-2 induced growth alterations and signaling pathways in NIH3T3 fibroblasts. Oncogene 16: 2819–2825. 9652750 10.1038/sj.onc.1201797

[25] Williams SP, Nowicki MO, Liu F, Press R, Godlewski J, Abdel-Rasoul M, et al (2011) Indirubins decrease glioma invasion by blocking migratory phenotypes in both the tumor and stromal endothelial cell compartments. Cancer Res 71: 5374–5380. 10.1158/0008-5472.CAN-10-3026 21697283 PMC4288480

[26] He TC, Sparks AB, Rago C, Hermeking H, Zawel L, da Costa LT, et al (1998) Identification of c-MYC as a target of the APC pathway. Science 281: 1509–1512. 9727977 10.1126/science.281.5382.1509

[27] Wang Z, Iwasaki M, Ficara F, Lin C, Matheny C, Wong SH, et al (2010) GSK-3 promotes conditional association of CREB and its coactivators with MEIS1 to facilitate HOX-mediated transcription and oncogenesis. Cancer Cell 17: 597–608. 10.1016/j.ccr.2010.04.024 20541704 PMC2919232

[28] Doble BW, Patel S, Wood GA, Kockeritz LK, Woodgett JR (2007) Functional redundancy of GSK-3alpha and GSK-3beta in Wnt/beta-catenin signaling shown by using an allelic series of embryonic stem cell lines. Dev Cell 12: 957–971. 17543867 10.1016/j.devcel.2007.04.001 PMC4485918

[29] Yadav V, Zhang X, Liu J, Estrem S, Li S, Gong XQ, et al (2012) Reactivation of Mitogen-Activated Protein Kinase (MAPK) Pathway by FGF Receptor 3 (FGFR3)/Ras Mediates Resistance to Vemurafenib in Human B-RAF V600E Mutant Melanoma. J Biol Chem. 10.1074/jbc.M112.377218 PMC3431627 22730329

[30] Yamanaka H, Moriguchi T, Masuyama N, Kusakabe M, Hanafusa H, Takada R, et al (2002) JNK functions in the non-canonical Wnt pathway to regulate convergent extension movements in vertebrates. EMBO Rep 3: 69–75. 11751577 10.1093/embo-reports/kvf008 PMC1083927

[31] Nateri AS, Spencer-Dene B, Behrens A (2005) Interaction of phosphorylated c-Jun with TCF4 regulates intestinal cancer development. Nature 437: 281–285. 16007074 10.1038/nature03914

[32] Biechele TL, Kulikauskas RM, Toroni RA, Lucero OM, Swift RD, James RG, et al (2012) Wnt/beta-catenin signaling and AXIN1 regulate apoptosis triggered by inhibition of the mutant kinase BRAFV600E in human melanoma. Sci Signal 5: ra3. 22234612 10.1126/scisignal.2002274 PMC3297477

