# Supplementary material for: Activating the Wnt/β-Catenin Pathway for the Treatment of Melanoma – Application of LY2090314, a Novel Selective Inhibitor of Glycogen Synthase Kinase-3
Source: PLoS One. 2015 Apr 27;10(4):e0125028. doi: 10.1371/journal.pone.0125028 (PMC4411090; doi:10.1371/journal.pone.0125028)
Supplement: S1 Fig — Single point (20uM) percentage inhibition studies were performed for 200 kinases and IC50 experiments conducted for 44 enzymes. Fold selectivity relative to GSK3β is represented in the figure. (PDF) [file pone.0125028.s001.pdf]

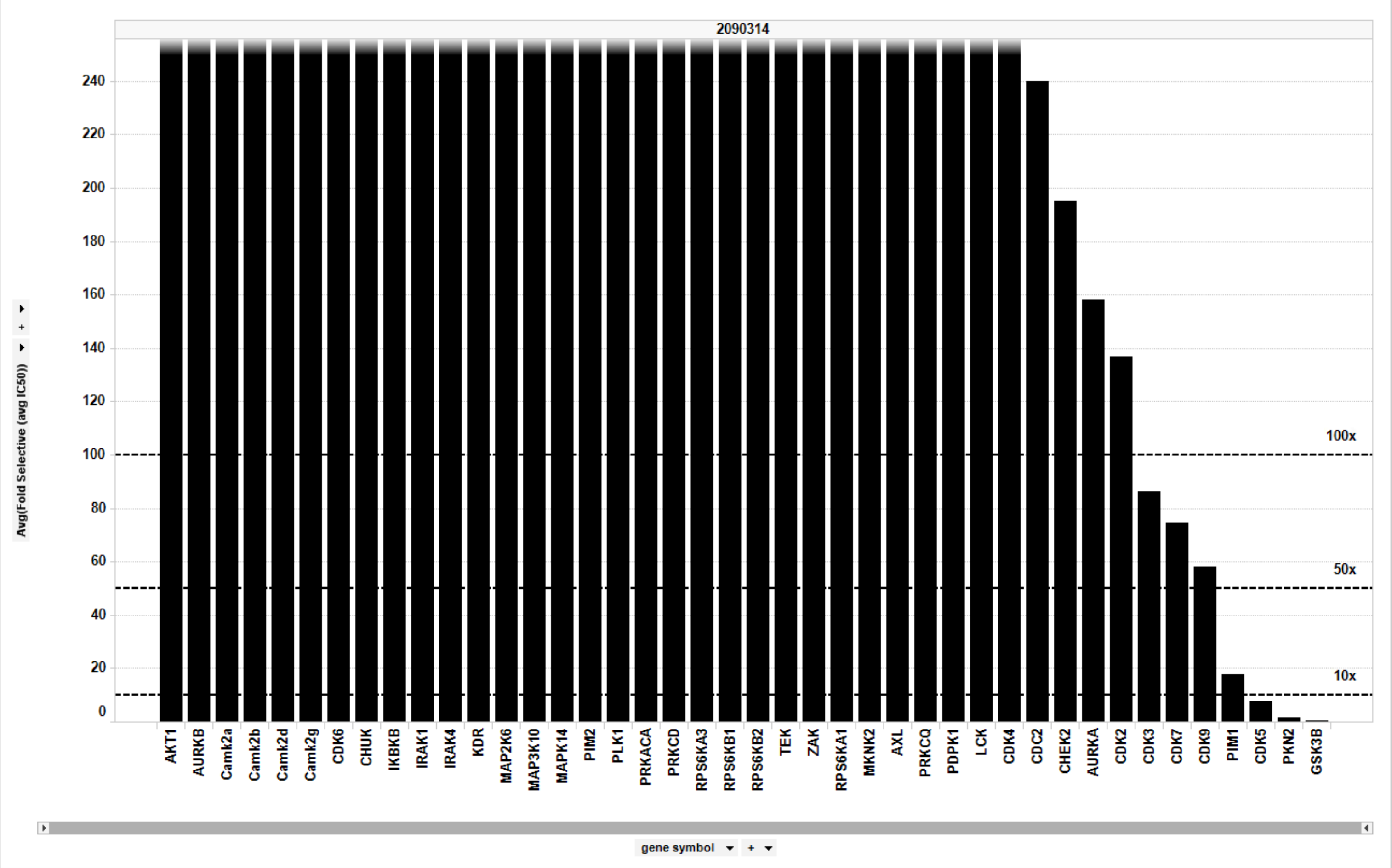

Figure S1. LY2090314 selectivity profile. Single point (20uM) percentage inhibition studies were performed for 200 kinases and IC50 experiments conducted for 44 enzymes. Fold selectivity relative to GSK3β is represented in the figure.
