# Supplementary figures and images for: Activating the Wnt/β-Catenin Pathway for the Treatment of Melanoma – Application of LY2090314, a Novel Selective Inhibitor of Glycogen Synthase Kinase-3
Source: PLoS One. 2015 Apr 27;10(4):e0125028. doi: 10.1371/journal.pone.0125028 (PMC4411090; doi:10.1371/journal.pone.0125028)

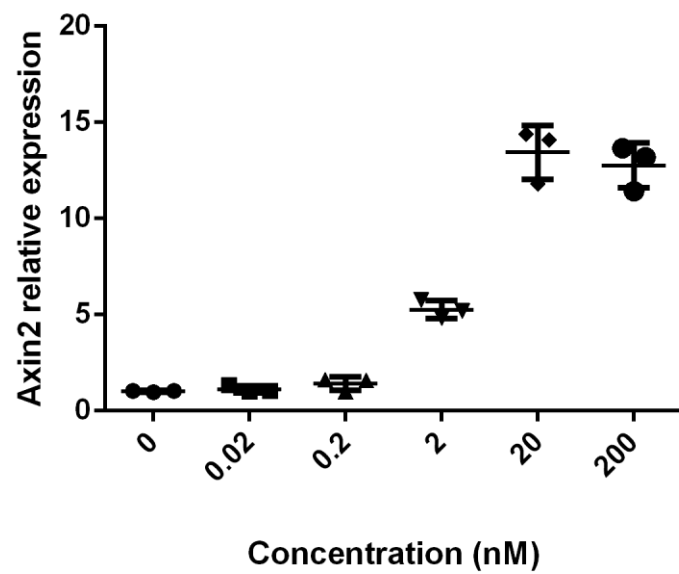

**Figure S2.** LY2090314 treatment of A375 cells increased Axin2 gene expression in a dose dependent manor.

Supplement: S2 Fig — (PDF) [file pone.0125028.s002.pdf]
