# Supplementary material for: Activating the Wnt/β-Catenin Pathway for the Treatment of Melanoma – Application of LY2090314, a Novel Selective Inhibitor of Glycogen Synthase Kinase-3
Source: PLoS One. 2015 Apr 27;10(4):e0125028. doi: 10.1371/journal.pone.0125028 (PMC4411090; doi:10.1371/journal.pone.0125028)
Supplement: S3 Fig — Dominant/negative TCF4 was delivered to cells using lentivirus. Following selection, cytotoxicity assays were performed with LY2090134 according to materials and methods (● A375 control; ■ A375 TCF4 DN; ▲ M14 control; ▼ M14 TCF4 DN). (PDF) [file pone.0125028.s003.pdf]

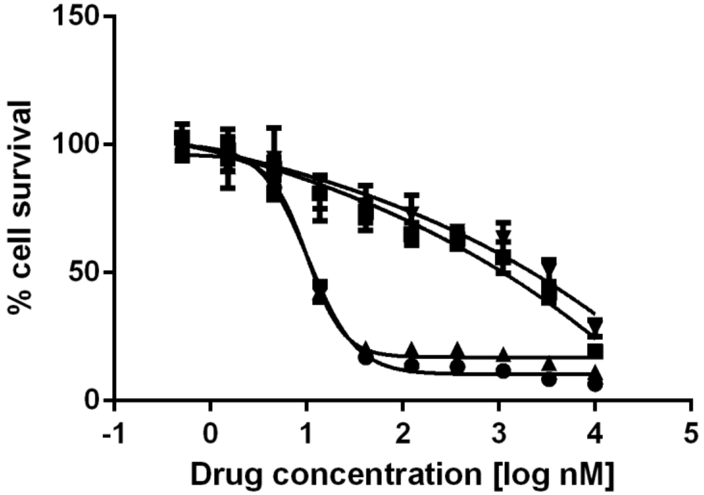

Figure S3. A dominant/negative TCF4 construct renders cells insensitive to LY2090314.. Dominant/negative TCF4 was delivered to cells using lentivirus. Following selection, cytotoxicity assays were performed with LY2090134 according to materials and methods (● A375 control; ■ A375 TCF4 DN; ▲ M14 control; ▼ M14 TCF4 DN).
