# Supplementary material for: Activating the Wnt/β-Catenin Pathway for the Treatment of Melanoma – Application of LY2090314, a Novel Selective Inhibitor of Glycogen Synthase Kinase-3
Source: PLoS One. 2015 Apr 27;10(4):e0125028. doi: 10.1371/journal.pone.0125028 (PMC4411090; doi:10.1371/journal.pone.0125028)
Supplement: S4 Fig — Following drug treatment, cells were analyzed for compound effect on the Wnt and Ras pathways and demonstrate differential signaling pathway modulation. (PDF) [file pone.0125028.s004.pdf]

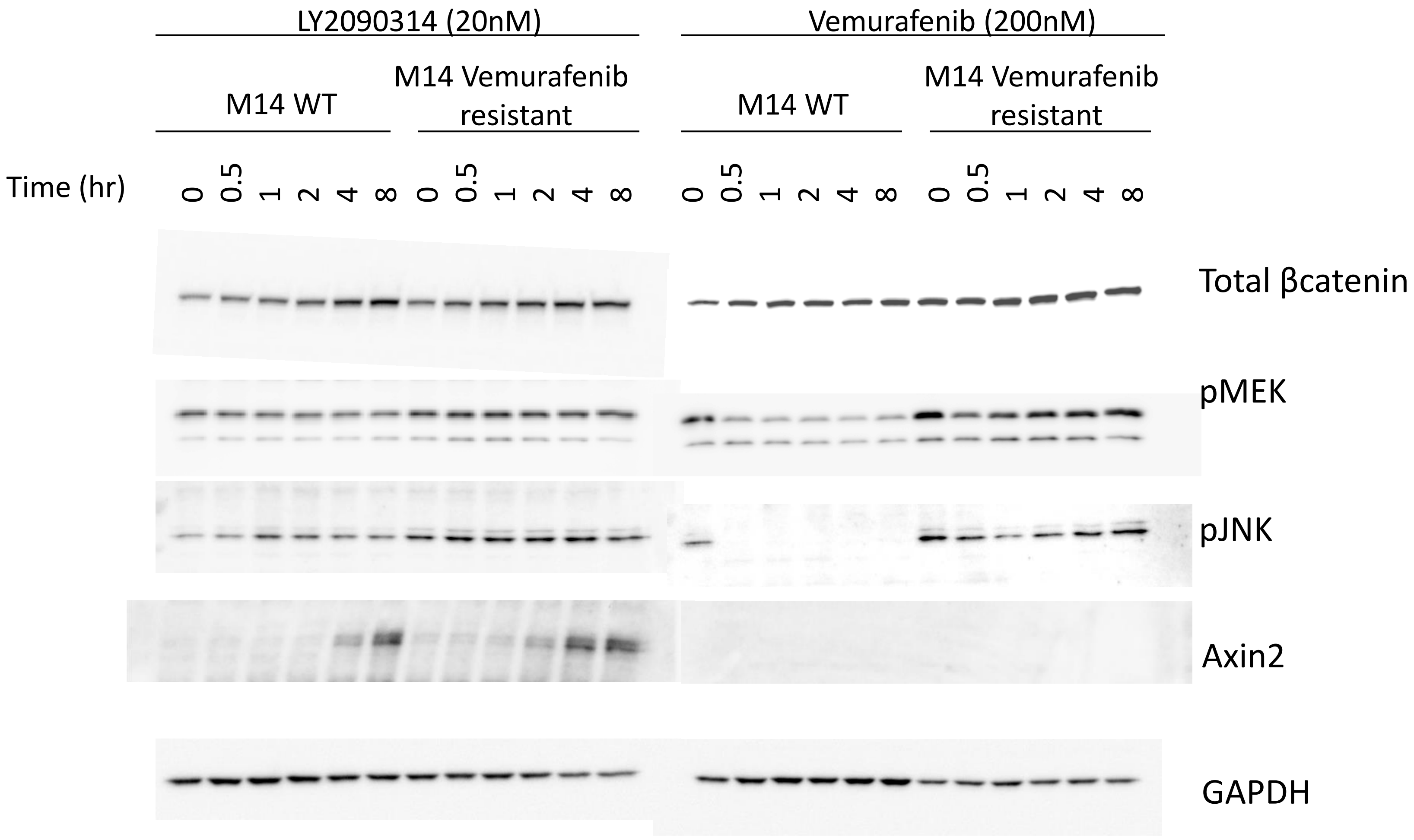


**Figure S4. LY2090314 and vemurafenib have distinct mechanisms of action.** Following drug treatment, cells were analyzed for compound effect on the Wnt and Ras pathways and demonstrate differential signaling pathway modulation**.**
