# Supplementary material for: Activating the Wnt/β-Catenin Pathway for the Treatment of Melanoma – Application of LY2090314, a Novel Selective Inhibitor of Glycogen Synthase Kinase-3
Source: PLoS One. 2015 Apr 27;10(4):e0125028. doi: 10.1371/journal.pone.0125028 (PMC4411090; doi:10.1371/journal.pone.0125028)
Supplement: S5 Fig — A. Melanoma cells stably transfected with shRNAs targeting Axin1 display decreased Axin1 protein expression by western blot. A375 (B) and M14 (C) cells expressing shRNAs targeting Axin1(● Control; ■ Axin1 shRNA 1; ▲ Axin1 shRNA 2; ▼ Axin1 shRNA 3) retain sensitivity to LY2090314 suggesting Axin1 does not play a role in the apoptotic response to compound treatment. (PDF) [file pone.0125028.s005.pdf]

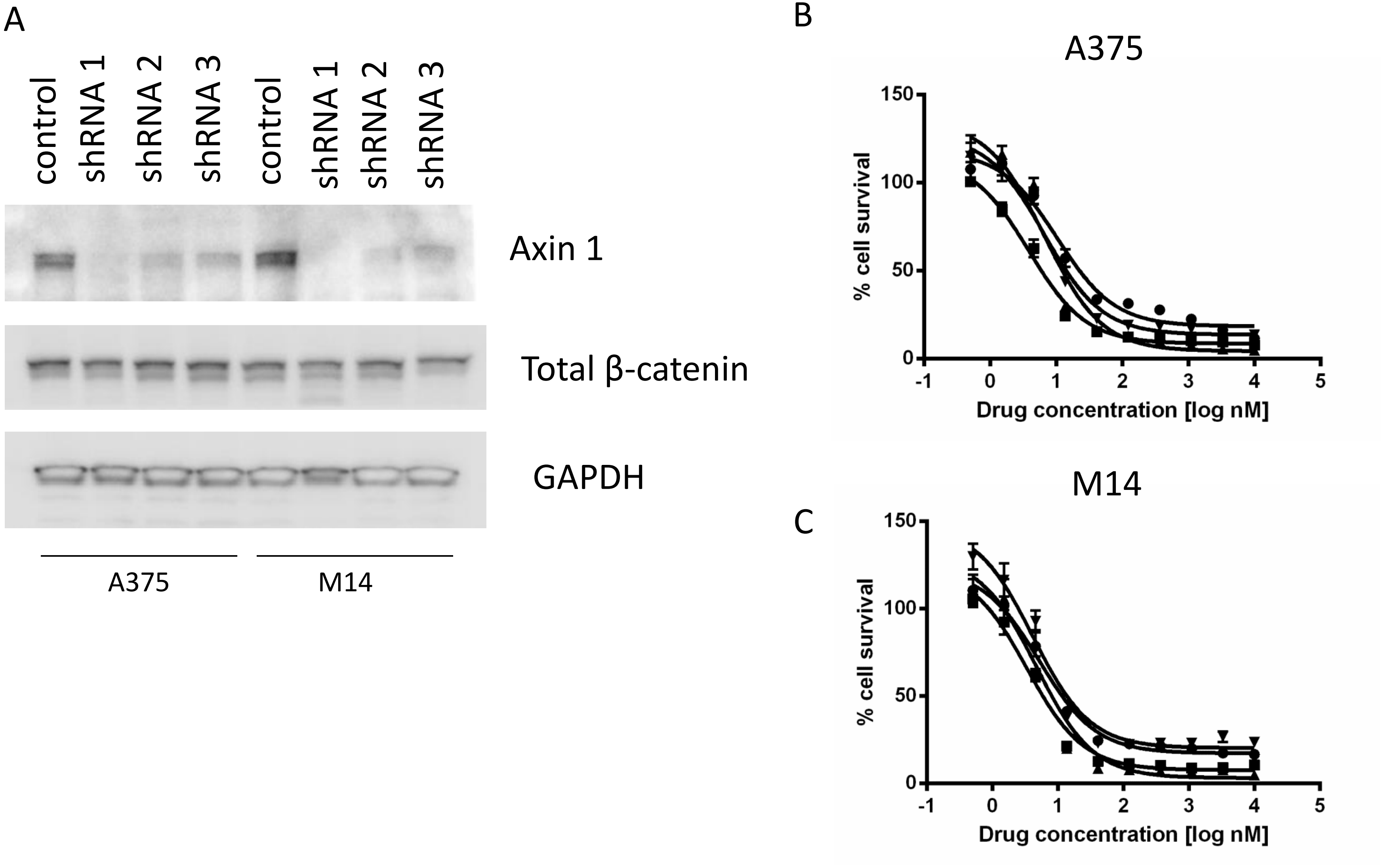

Figure S5. Cell death induced by LY2090314 is not dependent on Axin1. A. Melanoma cells stably transfected with shRNAs targeting Axin1 display decreased Axin1 protein expression by western blot. A375 (**B**) and M14 (**C**) cells expressing shRNAs targeting Axin1(● Control; ■ Axin1 shRNA 1; ▲ Axin1 shRNA 2; ▼ Axin1 shRNA 3) retain sensitivity to LY2090314 suggesting Axin1 does not play a role in the apoptotic response to compound treatment.
