# Supplementary material for: Activating the Wnt/β-Catenin Pathway for the Treatment of Melanoma – Application of LY2090314, a Novel Selective Inhibitor of Glycogen Synthase Kinase-3
Source: PLoS One. 2015 Apr 27;10(4):e0125028. doi: 10.1371/journal.pone.0125028 (PMC4411090; doi:10.1371/journal.pone.0125028)
Supplement: S6 Fig — Melanoma cells stably transfected with shRNAs targeting β-catenin display decreased β-catenin protein expression by western blot (Fig 3). A375 (A) and M14 (B) cells expressing shRNAs targeting β-catenin(● Control; ■ β-catenin shRNA 1; ▲ β-catenin shRNA 2; ▼ β-catenin shRNA 3) retain sensitivity to vemurafenib suggesting β-catenin does not play a role in the apoptotic response to compound treatment. (PDF) [file pone.0125028.s006.pdf]

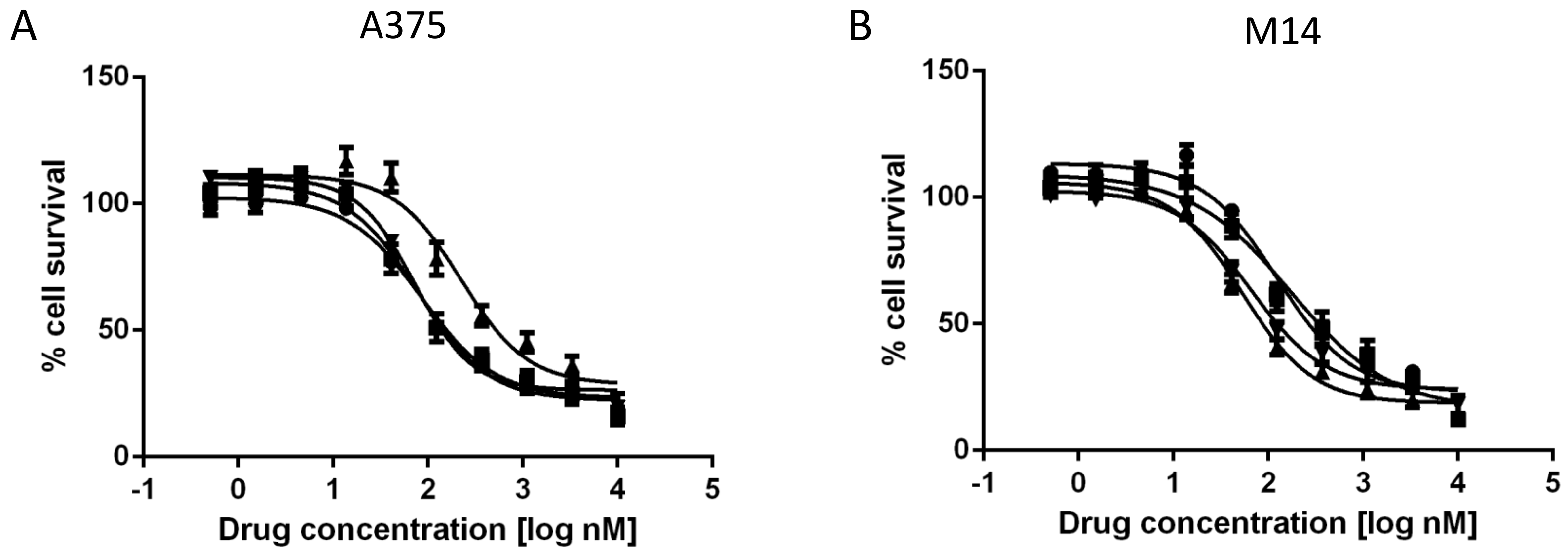

Figure S6. Cell death induced by Vemurafenib is not dependent on β-catenin. Melanoma cells stably transfected with shRNAs targeting β-catenin display decreased β-catenin protein expression by western blot (Figure 3). A375 (**A**) and M14 (**B**) cells expressing shRNAs targeting β-catenin(● Control; ■ β-catenin shRNA 1; ▲ β-catenin shRNA 2; ▼ β-catenin shRNA 3) retain sensitivity to vemurafenib suggesting β-catenin does not play a role in the apoptotic response to compound treatment.
