# Supplementary material for: Activating the Wnt/β-Catenin Pathway for the Treatment of Melanoma – Application of LY2090314, a Novel Selective Inhibitor of Glycogen Synthase Kinase-3
Source: PLoS One. 2015 Apr 27;10(4):e0125028. doi: 10.1371/journal.pone.0125028 (PMC4411090; doi:10.1371/journal.pone.0125028)
Supplement: S1 Table — Cell lines were treated for 72 hours in an exponential phase of growth and the IC50 determined using a cell viability method, CellTiter-Glo (see materials and methods). (PDF) [file pone.0125028.s007.pdf]

Cell line	Relative IC50 (µM)	Tumor type
HCT116	10.48	Colorectal adenocarcinoma
COLO205	11.30	
RKO	>20	
COLO320	>20	
A549	9.96	Lung carcinoma
H2030	9.44	
H460	13.25	Large cell lung carcinoma
SK-MES-1	>20	Squamous cell lung carcinoma
SKBR3	>20	Breast adenocarcinoma
MDA-MB-231	>20	
MDA-MB-468	0.45	
MCF7	>20	
BxPC3	0.43	Prostate adenocarcinoma
LNCaP	0.15	
DU145	5.52	

Table S1. LY2090134 relative IC50 values in a panel of human tumor cell lines . Cell lines were treated for 72 hours in an exponential phase of growth and the IC50 determined using a cell viability method, CellTiter-Glo (see materials and methods).
